# Syringic acid from rice roots inhibits soil nitrification and N_2_O emission under red and paddy soils but not a calcareous soil

**DOI:** 10.3389/fpls.2022.1099689

**Published:** 2022-12-20

**Authors:** Yufang Lu, Yao Hua, Na Lv, Weijun Zu, Herbert J. Kronzucker, Gangqiang Dong, Weiming Shi

**Affiliations:** ^1^ State Key Laboratory of Soil and Sustainable Agriculture, Institute of Soil Science, Chinese Academy of Sciences, Nanjing, China; ^2^ University of Chinese Academy of Sciences, Beijing, China; ^3^ School of BioSciences, The University of Melbourne, Parkville, VIC, Australia; ^4^ Faculty of Land and Food Systems, University of British Columbia, Vancouver, BC, Canada; ^5^ Amway Botanical R&D Center, Wuxi, China

**Keywords:** biological nitrification inhibitor, syringic acid, ammonia-oxidizing bacteria, ammonia-oxidizing archaea, nitrous oxide

## Abstract

Syringic acid (SA) is a novel biological nitrification inhibitor (BNIs) discovered in rice root exudates with significant inhibition of *Nitrosomonas* strains. However, the inhibitory effect of SA on nitrification and nitrous oxide (N_2_O) emissions in different soils and the environmental factors controlling the degree of inhibition have not been studied. Using 14-day microcosm incubation, we investigated the effects of different concentrations of SA on nitrification activity, abundance of ammonia-oxidizing microorganisms, and N_2_O emissions in three typical agricultural soils. The nitrification inhibitory efficacy of SA was strongest in acidic red soil, followed by weakly acidic paddy soil, with no significant effect in an alkaline calcareous soil. Potential nitrification activity (PNA) were also greatly reduced by SA additions in paddy and red soil. Pearson correlation analysis showed that the inhibitory efficacy of SA might be negatively correlated with soil pH and positively correlated with clay percentage. SA treatments significantly reduced N_2_O emissions by 69.1-79.3% from paddy soil and by 40.8%-46.4% from red soil, respectively, but no effect was recorded in the calcareous soil. SA addition possessed dual inhibition of both ammonia-oxidizing archaea (AOA) and ammonia-oxidizing bacteria (AOB) abundance in paddy and red soil. Structural equation modelling revealed that soil ammonium (NH_4_^+^) and dissolved organic carbon content (DOC) were the key variables explaining AOA and AOB abundance and subsequent N_2_O emissions. Our results support the potential for the use of the BNI SA in mitigating N_2_O emissions and enhancing N utilization in red and paddy soils.

## Introduction

Ammonium (NH_4_^+^) is the main form of nitrogen (N) absorbed by plants (Kronzucker et al., 1997; Glass et al., 2002). NH_4_^+^ can also be readily oxidized by soil microbes, producing nitrite (NO_2_^-^) and nitrate (NO_3_^-^) through the process of nitrification, which leads to significant losses of N fertilizer, atmospheric N pollution caused by emissions of nitrous oxide (N_2_O), and NO_3_^-^ pollution of waterways (Coskun et al., 2017a; Coskun et al., 2017b). Inhibiting nitrification of NH_4_^+^ into NO_3_^-^ can reduce such N losses (Coskun et al., 2017a; Coskun et al., 2017b), an approach also recently proposed as a more generalized “ammonium solution” to reduce N pollution from agricultural fields and to enhance crop yield (Subbarao and Searchinger, 2021). Practices such as deep N-fertilizer placement and controlled-release fertilizers have also been proposed to stabilize reduced N in soils and minimize N conversion and losses (Zheng et al., 2016; Min et al., 2021a). While the application of several synthetic nitrification inhibitors (SNIs) has increased N utilization in fields (Zaman et al., 2009; Min et al., 2021b), limitations such as high cost, inconsistency in field performance, inability to function in acidic environments, and food safety risks have prevented their widespread adoption in modern agriculture (Subbarao et al., 2012).

The use of plant-derived biological nitrification inhibitors (BNIs) is an environmentally friendly strategy to reduce N pollution and boost crop yields (Subbarao et al., 2012; Coskun et al., 2017b). BNIs have the potential to overcome the limitations of SNIs through breeding crop varieties with higher BNI capacity (Subbarao and Searchinger, 2021). While BNI capacity has been well evaluated in tropical pasture plants, field crops, and trees (O'Sullivan et al., 2016; Laffite et al., 2020), relatively less is known about BNIs in the cereal crops, rice, wheat, and maize (Coskun et al., 2017b). In previous studies, we reported the first BNI 1,9-decanediol (a hydrophobic fatty alcohol) from root exudates of rice (Sun et al., 2016; Zhang et al., 2019). Subsequently, a second BNI exuded from roots of cultivated rices, syringic acid (SA, a hydrophilic phenolic acid), was discovered, which displayed synergism with 1,9-decanediol in inhibiting nitrification carried out by soil microorganisms (Lu et al., 2022).

Compared with research on SNIs, research on BNIs is still in its infancy. A small number of recent studies have focused on the nitrification-inhibitory effect of some BNIs in field soils when applied as pure compounds. Methyl 3,4-hydroxyphenyl propionate (MHPP) from sorghum roots was shown to suppress nitrification in a neutral soil, whereas the hydrophilic sakuranetin had no inhibitory effect (Subbarao et al., 2013). A recent study by Ma et al. (2021) demonstrated that two long-chain unsaturated fatty acids, linoleic acid (LA) and linolenic acid (LN), from the shoot tissue of pasture grass can cause nitrification inhibition in an acidic sandy loam. However, most of these BNI function tests have been verified in one type of soil at a time. Thus, it is necessary to assess the inhibitory profiles of BNIs on different soil types colonised by different ammonia-oxidizing microorganisms, as it will help identify precise targets and the range of possible applications of BNIs in agricultural N management.

The efficacies of SNIs have been extensively studied and can be highly variable across soils. These differences in efficacy have been ascribed to differences in soil pH, water content, temperature, organic matter content, clay percentage, and applied NI dose (Barth et al., 2008; Shi et al., 2016; Guardia et al., 2018; Cui et al., 2021). Only two soil-based incubation studies have thus far evaluated the efficacy of a BNI compound in different soil types. Lu et al. (2019) showed that 1,9-decanediol, exuded by rice roots, can act as a more potent BNI in acidic soil than in alkaline soil, underscoring that the inhibition profile of BNIs varies with soil pH and free BNI concentration. The nitrification inhibition of MHPP was higher in the acidic soil than in the calcareous soil (Lan et al., 2022). For the recently identified phenolic BNI SA from rice root exudates, the inhibition profile on different soils, and the key factors responsible for differences in inhibition profile, have not been examined.

BNIs are considered a “green” and cost-effective strategy to mitigating agricultural greenhouse gas emissions (Subbarao et al., 2017). In addition to planting tropical forage grasses or sorghum with high BNI capacity (Subbarao et al., 2009; Zhang et al., 2015; Byrnes et al., 2017; Villegas et al., 2020), several recent studies have evaluated the potential role of direct application of specific BNI compounds in reducing soil N_2_O emission. The fatty alcohol 1,9-decanediol obtained from rice root exudates was shown to significantly reduce N_2_O emissions by an average of 48% in three agricultural soils (Lu et al., 2019). N_2_O emissions could be reduced by >60% when the phenylpropanoid MHPP was combined with other N-management measures such as root-zone fertilization, the application of urease inhibitors, or that of biochar (Yao et al., 2020; Lan et al., 2021). However, Ma et al. (2021) have pointed out the risk of promoting N_2_O emissions by the addition of high concentrations of two fatty acids, LN and LA. Thus, not all BNIs are actually effective in mitigating N_2_O emissions, and the efficacy of BNIs to reduce N_2_O emissions may depend on BNI type. It remains unknown whether the newly-discovered phenolic acid SA can inhibit N_2_O emission in different soils.

As drivers of the first and rate-limiting step of nitrification, ammonia-oxidizing archaea (AOA) and ammonia-oxidizing bacteria (AOB) are considered the principal microbial contributors to N_2_O emissions (Santoro et al., 2011; Wang et al., 2015). Due to different metabolic pathways, AOB and AOA are likely to occupy different niches across soils, driven by soil pH, temperature, dissolved organic carbon and soil N level (Prosser et al., 2020; Tao et al., 2021a; Tao et al., 2021b). BNIs have the potential to regulate both the AOA and AOB community (Nardi et al., 2013; Lu et al., 2019; Sarr et al., 2020; Lan et al., 2022). However, changes in ammonia oxidizer communities are only contributory to nitrification, and the relationship to subsequent N_2_O emissions and the relevant abiotic control factors have not been characterised.

To better understand different soil types where SA acts as an inhibitor of nitrification and N_2_O emission, 14-day microcosm experiments were conducted in three agricultural soils with varing properties. Different amounts of SA were applied to monitor the nitrogen dynamics, the abundance of ammonia oxidizers, and N_2_O emissions. The objectives were: (1) to explore the nitrification inhibitory impact of SA in different types of soil and relevant control factors, (2) to evaluate the potential of SA to reduce N_2_O emissions from soils, (3) to assess the effect of SA on the population of ammonia oxidizers, and (4) to establish the linkages between soil physicochemical properties, abundance of ammonia oxidizers, and N_2_O emissions by structural equation modeling (SEM).

## Materials and methods

### Soil sampling

Soil samples were collected from three sites, which represent the calcareous soil, paddy soil, and red soil. The calcareous soil (sandy loam) was collected from Dezhou (36°83′ N, 116°58′ E), a paddy soil (silt loam) was collected from Yinxin (31°39′ N, 119°28′ E), and the red soil (loamy clay) was sampled from Yintan (26°45′ N, 111°52′ E), which are located in typical agricultural areas of China. Soil samples (0–20 cm depth) were collected, air-dried, and sieved through 2-mm mesh before use.

### Soil physicochemical analysis

Soil pH was measured by fresh soil (1:2.5 (w/v) soil to water solution) using pH electrodes (Mettler Toledo, Switzerland). Soil exchangeable NH_4_^+^-N and NO_3_^−^-N were colorimetrically quantified in 2 mol L^-1^ KCl extracts by continuous flow analysis (Skalar, Breda, Netherlands). Total C and total N were detected using a Vario Max CN analyzer (Elementar, Hanau, Germany). Soil organic matter (SOM) was determined following the K_2_Cr_2_O_7_ wet oxidation method. Soil texture (sand, silt, and clay fractions) was assessed with a laser diffraction particle size analyzer (LS13320, Beckman Coulter Co.). The dissolved organic carbon (DOC) concentration was determined by a TOC analyzer (Multi N/C 3100, Analytik Jena AG). Details of three soil physicochemical characteristics are shown in **Table 1**.

**Table 1:** Physicochemical characteristics of the tested three soils.

Soil type	Calcareous soil	Paddy soil	Red soil
Soil pH	8.16	6.14	4.49
Organic matter (%)	1.07	1.87	1.16
NH_4_^+^-N (mg kg^-1^)	3.48	15.04	3.89
NO_3_^-^-N (mg kg^-1^)	7.23	7.93	10.31
Total C (%)	1.64	1.03	0.62
Total N (%)	0.040	0.113	0.068
Texture	Sandy loam	Silt loam	Loamy clay
Particle size (%)			
Sand (0.02-2 mm)	53.2	45.0	20.8
Silt (0.002-0.02 mm)	36.4	39.2	23.2
Clay (<0.002 mm)	10.4	15.8	56.0

### Soil microcosm experiments

The laboratory soil incubation was set up in 125-ml serum vials containing 20 g of soils (oven dry-weight equivalent). SA (C_9_H_10_O_5_; MW:198) and dicyandiamide (DCD) were purchased from Sigma-Aldrich (Shanghai, China). The five treatments were performed in triplicate: 1) (NH_4_)_2_SO_4_ control (N 200 mg kg^−1^ soil, CK); 2) (NH_4_)_2_SO_4_ plus SA at 500 mg kg^−1^ soil (SA-high dose, SA-500); 3) (NH_4_)_2_SO_4_ plus SA at 200 mg kg^−1^ soil (SA-medium dose, SA-200); 4) (NH_4_)_2_SO_4_ plus SA at 100 mg kg^−1^ soil (SA-low dose, SA-100); 5) (NH_4_)_2_SO_4_ plus DCD at 20 mg kg^−1^ soil (10% of applied NH_4_^+^-N according to the typically recommended rate, DCD) (McGeough et al., 2016). Inhibition by the biological nitrification inhibitor SA was compared with that by the synthetic nitrification inhibitor DCD, to gain a better understanding of how various soil types respond to different inhibitor types. The dosages of SA were chosen according to our previous study where the nitrification inhibitory efficacy was highest at 500 mg kg^-1^ and smallest at 100 mg kg^-1^ (Lu et al., 2022). These dosages also fall into the range of BNI application rates in other soil incubation experiments (Subbarao et al., 2008; Nardi et al., 2013; Subbarao et al., 2013; Ma et al., 2021). The SA powder was dissolved in (NH_4_)_2_SO_4_ solution by ultrasound exposure, the solution was then applied uniformly to soils according to Lu et al. (2019). The vials were incubated at 60% waterfilled pore space (WFPS) in the dark in a temperature-controlled incubator at 25°C. Every three days, the bottles were opened for aeration and weighed, and then the appropriate amount of deionised water was added for maintaining soil moisture. Soil samples were destructively collected on 0, 7, 14 days of incubation. Potential nitrification activity (PNA) was determined *via* the shaken slurry method described by Hart et al. (1994) and the details were given in Supplementary materials. Nitrification inhibitory efficacy (NIE, %) was calculated using the following formula, according to Lu et al. (2019): Nitrification inhibitory efficacy (NIE, %) = ((NO_3_^−^-N produced in the (NH_4_)_2_SO_4_ control) – (NO_3_^−^-N produced in the SA and DCD treatments))/(NO_3_^−^-N produced in the (NH_4_)_2_SO_4_ control) × 100.

### Gas sampling and N_2_O flux measurement

After 24 h of closure, gas samples (5 mL) from the headspace using syringe (20 mL) were collected at 1, 2, 3, 4, 5, 7, 10 and 14 days, and were transferred to pre-evacuated 20-mL headspace gas containers. After sampling, all bottles were ventilated for 30 min and then resealed. Gas samples were determined for N_2_O concentrations by gas chromatograph (HP7820A, Agilent Technologies, CA, USA) equipped with an electron capture detector (ECD).

The N_2_O fluxes were calculated according to Tao et al. (2021b), using the equation described below:


$$F=\frac{dc}{dt}\;\frac{M}{Vm}V\frac{273}{273+T\;}\frac{1}{m}$$

where F: N_2_O emission flux, μg kg^−1^ soil d^−1^ (N_2_O-N); dc: gas concentration; dt: sampling interval; M: molar mass, 28 g mol^−1^ (N_2_O-N); Vm: molar volume of gas, 22.4 L mol^−1^; V: headspace of the bottle, L; T: incubation temperature,°C; m: soil dry weight, kg.

Cumulative N_2_O emissions (E, mg N_2_O-N kg^-1^ soil) were calculated according to Tao et al. (2021b), using the following equation:


$$EN_{2}O=\sum^{}[(F_{n+1}+F_{n})/2]\times \left(D_{n+1}-D_{n}\right)/1000$$

where F represents the N_2_O flux (μg N_2_O-N kg^−1^ soil d^−1^), n is the n^th^ sampling, and (D_n+1_-D_n_) represents the number of days between two adjacent samplings.

### Soil DNA extraction and quantitive PCR analysis

DNA was extracted from 0.25 g of freeze-dried soil using MoBio PowerSoil DNA-isolation kits (MoBio Laboratories, Carlsbad, CA, USA). The purity and quantity of the extracted DNA were determined by a NanoDrop ND1000 spectrophotometer (NanoDrop Technologies, Wilmington, USA) and the samples were stored at −20°C until use.

Real-time quantitative PCR (qPCR) was performed to quantify the abundance of AOA and AOB ammonia monooxygenase genes (*amoA*). The PCR assays were conducted on a LightCycler 480 (Roche Diagnostics, Mannheim, Germany), using the primer pairs Arch-amoAF/Arch-amoA (Francis et al., 2005) and amoA-1F/amoA-2R (Rotthauwe et al., 1997), respectively. The 10-μL reaction mixture contained 5 μL of SYBR Premix Ex Taq (TaKaRa, Tokyo, Japan), 0.4 μL of each of the forward and reverse primers (10 μM), and 0.5 μL of dilluted DNA as a template. Standard curves dilution series from 1×10^1^ to 1×10^7^ copies were created. qPCR was conducted in triplicate, and amplification efficiencies ranged from 86.2–94.6%, with R^2^ values > 0.99.

### Statistical analysis

One-way ANOVA was applied in SPSS Statistics 18.0 to determine the effect of SA on soil NH_4_^+^-N and NO_3_^−^-N, PNA, cumulative N_2_O emissions, AOA and AOB abundance. In addition, soil pH, SOM, clay percentage, PNA, and percent nitrification inhibitory efficacy among the three soils were analyzed by using the Pearson correlation test with Origin 2022. A linear regression analysis was used to study the relationship between soil NO_3_^−^-N and the abundance of ammonia oxidizers using Origin 2022. SEM was conducted to explore the causal linkages among N_2_O emissions AOA and AOB abundance, and soil properties, using the software AMOS 22.0. Several indicators were used to evaluate the overall fit of the model, ie., the *P* value, Chi-square value (χ^2^), comparative fit index (CFI), and root mean square error of approximation (RMSEA).

## Results

### NO_3_^–^-N, NH_4_^+^-N concentrations

**Figure 1:**
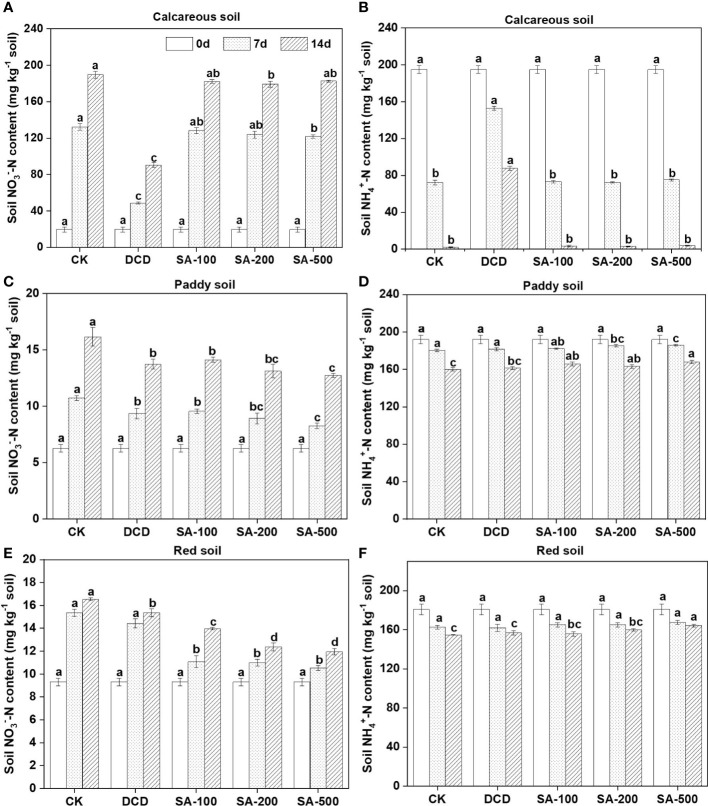
Effect of different concentrations of syringic acid (SA) and dicyandiamide (DCD) on soil NO_3_^-^ and NH_4_^+^ concentrations in the calcareous **(A, B)**, paddy **(C, D)**, and red soil **(E, F)** during a 14-d incubation. Values are means ± SE (n=3). Different letters indicate significant differences at *P* < 0.05 (LSD test) among treatments at each sampling time.

In the (NH_4_)_2_SO_4_ control of the calcareous soil, the NO_3_^–^-N concentrations showed an increasing trend from 19.7 mg kg^-1^ soil to 189.6 mg kg^-1^ at day 14 (**Figure 1A**). The NH_4_^+^-N contents decreased rapidly from 195 mg kg^-1^ soil to 72.5 mg kg^-1^ soil during the first 7 days, and showed further reduction to 2.25 mg kg^-1^ soil at day 14 (**Figure 1B**). There was no evidence of significant inhibition by SA at all three dose treatments, and the concentrations of soil NH_4_^+^-N and NO_3_^–^-N remained unchanged both at day 7 and day 14 compared to the control. By contrast, DCD addition significantly (*P* < 0.05) decreased the formation of NO_3_^–^-N (**Figure 1A**) and slowed the NH_4_^+^-N oxidation down at two sampling points, with about 87.7 mg NH_4_^+^-N kg^-1^ soil remaining in the end (**Figure 1B**).

Although the nitrification rate of the weakly acidic paddy soil was less than that of the alkaline calcareous soil, low-dose, medium-dose, and high-dose applications of SA all significantly inhibited nitrate production at day 7 by 10.9%, 16.7%, and 23.0%, respectively compared to the control (**Figure 1C**). This is in line with the higher NH_4_^+^-N level compared to the control (**Figure 1D**). Similarly, the addition of DCD slowed down the formation of NO_3_^–^-N by 12.8% compared to the control, but the NO_3_^–^-N amount was higher than that in the high-dose SA treatment at the end of incubation (**Figure 1C**), suggesting a weaker inhibition of DCD than of SA in the paddy soil.

In the acidic red soil, there was no apparent dose-response relationship between SA and nitrification inhibitory efficacy (**Figures 1E, F**). Compared to the N control, the low-dose, medium-dose, and high-dose applications of SA significantly inhibited nitrate production by 27.8%, 28.4%, and 31.4% at day 7, respectively (**Table 2**), whereas DCD showed no significant inhibition. Moreover, the inhibition by SA in the red soil was persistent and superior to that in the other two soils during the 14-day incubation.

**Table 2:** Nitrification inhibitory efficacy (%) by SA and DCD treatments among three agricultural soils at day 7 and day 14.

Treatments	Nitrification inhibitory efficacy %					
	Calcareous soil		Paddy soil		Red soil	
	7d	14d	7d	14d	7d	14d
SA-100	3.0 ± 2.4 b	4.0 ± 1.1 b	10.9 ± 1.8 b	12.6 ± 1.5 b	27.8 ± 3.3 a	15.6 ± 0.7 b
SA-200	6.3 ± 2.8 b	5.5 ± 1.7 b	16.7 ± 4.3 ab	18.9 ± 3.6 ab	28.4 ± 2.0 a	25.3 ± 2.1 a
SA-500	8.0 ± 1.3 b	3.6 ± 0.5 b	23.0 ± 2.3 a	21.2 ± 1.1 a	31.4 ± 1.3 a	27.8 ± 1.7 a
DCD	63.1 ± 0.8 a	52.2 ± 1.5 a	12.8 ± 4.3 b	15.1 ± 2.7 b	6.1 ± 2.6 b	7.2 ± 2.1 c

Values are means ± SE (n=3). Different letters indicate significant differences at P < 0.05 (LSD) among treatments at each sampling time for each soil type.

### Potential nitrification activity

**Figure 2:**
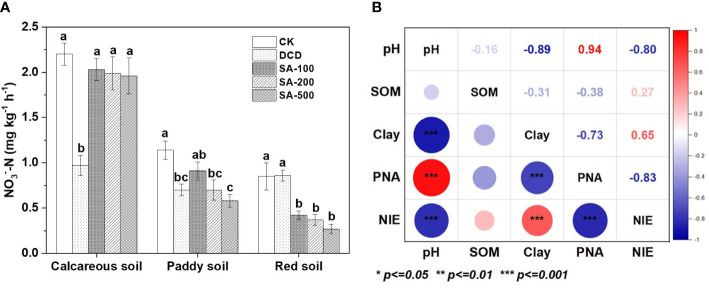
The potential nitrification activity (PNA) affected by different treatments in three soils at the end of incubation **(A)**, and the Pearson correlation test between soil properties (pH, soil organic matter (SOM), and clay percentage), PNA, and nitrification inhibitory efficacy (NIE, %) **(B)**. the Pearson correlation test between soil properties (pH, soil organic matter (SOM), and clay percentage), PNA, and nitrification inhibitory efficacy (NIE, %)**(B)**. Values are means ± SE (n=3). Different letters indicate significant differences at *P* < 0.05 (LSD test) among treatments at each soil type. The sizes of the circles and the shades of color represent the degree of relevance. The numbers are the correlation coefficients.

To obtain more insight into the nitrification inhibitory spectrum of SA, the PNA of soil samples was examined during the incubation process. As can be seen from **Figure 2**, the PNA in the (NH_4_)_2_SO_4_ control was 2.2, 1.2 and 0.9 mg NO_3_^–^-N kg^-1^ h^-1^ in the calcareous, paddy and red soil, respectively. All SA treatments showed no significant effect on PNA in the calcareous soil, but a 55% reduction was found in the DCD treatment compared with the (NH_4_)_2_SO_4_ control. In the paddy soil, PNA decreased by 20-49% in soil samples treated with SA and with DCD as compared to the control, but with no significant difference between the SA and DCD treatments. In the red soil, SA treatments showed a lower PNA than in the other two soils, but no significant effect in the DCD treatment could be observed, indicating that the inhibition of SA was superior to DCD in red soil.

Soil PNA had a significantly positive association with soil pH (r = 0.94, *P* < 0.001), but it had a negative correlation with soil clay percentage (r = -0.73, *P* < 0.001) and nitrification inhibitory efficacy (%, NIE, r = -0.83, *P* < 0.001). This negative correlation between NIE and PNA further verifies the nitrification inhibitory function of SA. NIE by SA was shown to be negatively associated with soil pH (r = -0.80, *P* < 0.001), and positively correlated with clay percentage (r = 0.65, *P* < 0.001). There was no significant correlation between PNA, NIE, and clay percentage with soil SOM (**Figure 2B**). This indicates that the differences in inhibition of nitrification by SA among the three soils might be attributed to the physicochemical properties of soil pH and clay percentage.

### N_2_O emissions

**Figure 3:**
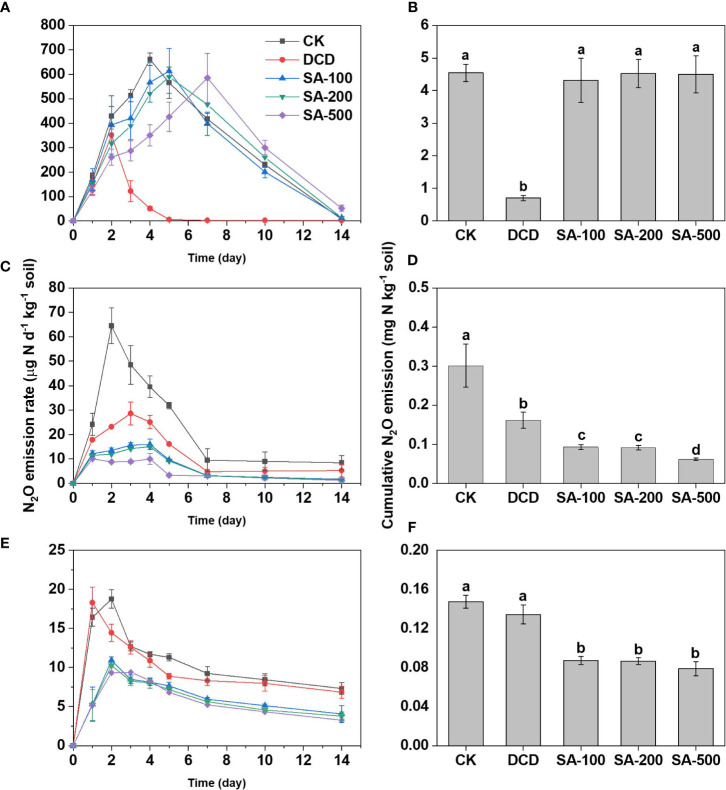
The dynamic change of N_2_O flux and cumulative emissions in calcareous soil **(A, B)**, paddy soil **(C, D)**, and red soil **(E, F)** during the 14-d incubation. Values are means ± SE (n=3). Different letters indicate significant differences at *P* < 0.05 (LSD test) among treatments.

The trends for soil N_2_O emission fluxes varied with soil type and treatment. Emissions in the (NH_4_)_2_SO_4_ control (CK) followed the order: calcareous soil > paddy soil > red soil. For the calcareous soil, N fertilizer addition produced an N_2_O emission peak of 661.4 μg N d^-1^ kg^-1^ soil at day 4 (**Figure 3A**). DCD strongly inhibited N_2_O emission, with an 82.2% lower accumulation than that in CK. Although SA treatments delayed the peak time for N_2_O generation, it didn’t significantly affect the cumulative N_2_O emissions (**Figure 3B**). In the paddy soil, the low-, medium and high-dose SA treatments not only reduced the peak N_2_O value (**Figure 3C**), but also suppressed the cumulative emission by 69.1%, 69.6% and 79.4% compared to the CK, respectively, which was significantly stronger than DCD’s 46.2% reduction (**Figure 3D**). Similarly, compared to the CK, low-, medium- and high-dose SA substantially inhibited N_2_O emission during the entire incubation period in red soil, resulting in a 40.8%, 41.3% and 46.4% reduction in cumulative N_2_O emissions, respectively, while DCD had no significant effect (**Figures 3E, F**).

### Abundance of AOB and AOA

**Figure 4:**
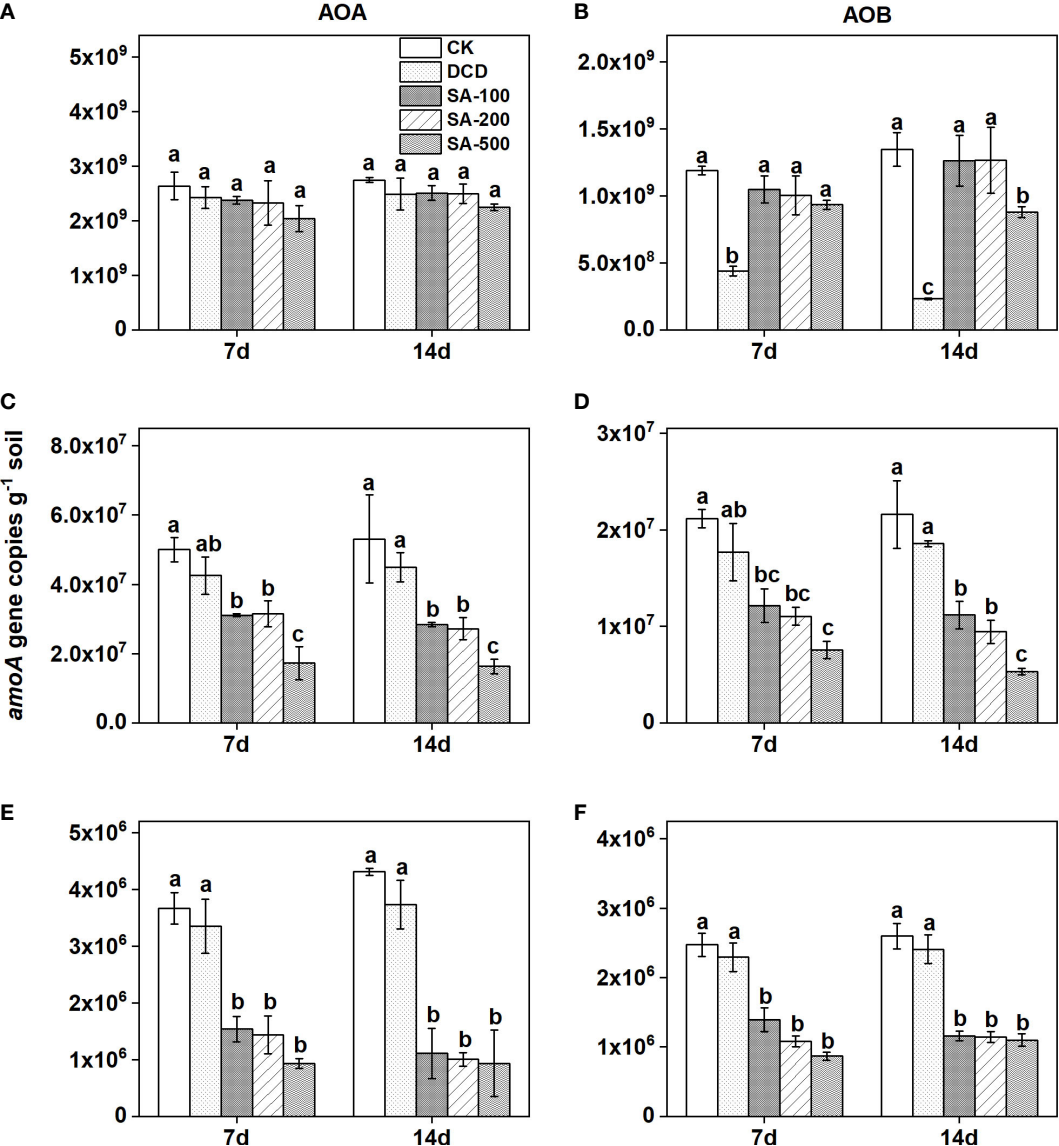
The *amoA* gene copy numbers of ammonia-oxidizing archaea (AOA) and bacteria (AOB) in calcareous soil **(A, B)**, paddy soil **(C, D)**, and red soil **(E, F)** under different treatments at day 7 and day 14 days. Values are means ± SE (n=3). Different letters indicate significant differences at *P* < 0.05 (LSD test) among treatments.

As compared with the control, no significant inhibition of different doses of SA addition was observed on the abundances of AOA and AOB in the calcareous soil both at day 7 and day 14, with the exception of high-dose SA for AOB at day 14 (**Figure 4B**). DCD treatment significantly (*P* < 0.05) decreased the abundance of AOB, by 63.1% and 82.7% at day 7 and 14 compared to the control, respectively, but it showed no significant inhibition on AOA abundance (**Figure 4A**).

In the paddy soil, AOA abundance significantly (*P* < 0.05) decreased, by 37.8%, 37.0%, and 65.5% at day 7, and by 46.5%, 48.7%, and 69.2% at day 14 in the presence of low-, medium-, and high-dose SA as compared with the control, respectively, but no significant inhibition of DCD could be found (**Figure 4C**). The abundance of AOB was also lower, by 16.4% at day 7 and by 14.1% at day 14, in the DCD treatment than in the control, and three SA addition treatments significantly reduced AOB abundance (*P* < 0.05), by 42.8-64.3% at day 7 and by 48.2-75.3% at day 14, relative to that in the control (**Figure 4D**).

Similar to the paddy soil, the AOA and AOB abundance were both significantly inhibited by all SA treatments in the red soil (**Figures 4E, F**). Meanwhile, AOA was shown to be more sensitive to SA than AOB. As compared with the control, the inhibition of AOA and AOB abundance by low-, medium-, and high-dose SA treatments reached 58.0-74.6% and 43.7-64.8% at day 7, and reached 74.3%-78.3% and 55.4%-60.2% at day 14, respectively. However, the *amoA* gene copies of AOA remained unchanged in the treatment of DCD (**Figure 4E**).

Significant and positive correlations were observed between AOB abundance and NO_3_^–^N contents in red soil (R^2^ = 0.44, *P* < 0.05), paddy soil (R^2^ = 0.54, *P* < 0.01), and calcareous soil (R^2^ = 0.68, *P* < 0.001) at day 14, while AOA abundance was positively associated with soil NO_3_^–^-N in paddy soil (R^2^ = 0.38, *P* < 0.01) and red soil (R^2^ = 0.59, *P* < 0.001), but not in calcareous soil (**Figure S1**).

### The relationships between soil properties, abundance of ammonia oxidizers, and N_2_O emissions

**Figure 5:**
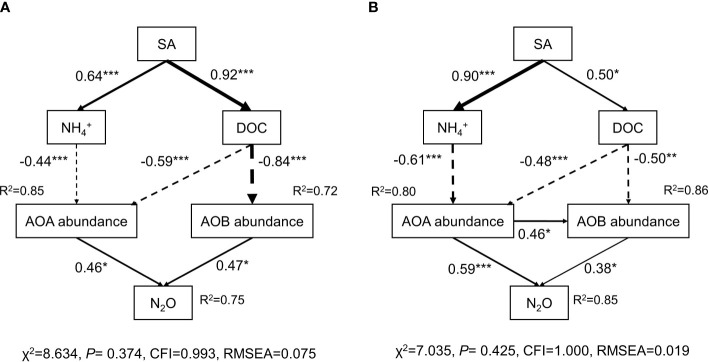
The structural equation model (SEM) explaining the mechanisms driving N_2_O emission as SA induces changes in soil properties and influences ammonia oxidizer communities in paddy and red soils. The arrow width indicates the strength of the standardized path coefficients. The solid line represents positive effects and the dashed line represents negative effects. Numbers on the arrows indicate significant standardized path coefficients (*p < 0.05; **p < 0.01; ***p < 0.001). R^2^ indicates the proportion of the variables explained by the factors.

Structural equation modeling (SEM) was applied to explore the microbial mechanisms underlying the mitigation of N_2_O emissions by SA in the paddy soil and red soil (**Figure 5**). The final model explained 75% and 85% of the variation in the N_2_O emissions in the paddy soil and red soil, respectively. SA application significantly reduced AOA gene abundance (explaining % = 85%, 80%) by altering NH_4_^+^ concentration (-0.44***, -0.61***) and DOC content (-0.59***, -0.48***), and reduced AOB (explaining % = 72%, 86%) by changing DOC (-0.84***, -0.50***) in paddy soil and red soil, respectively. Furthermore, the N_2_O emissions were positively correlated with AOA abundance (0.59***) and with AOB abundance (0.38*) in red soil (**Figure 5B**). In paddy soil, a positive association was also found between N_2_O emissions and AOA abundance (0.47*), and AOB abundance (0.46*) (**Figure 5A**). SEM results indicate that SA application mitigates N_2_O emissions by directly changing the soil-environmental factors of NH_4_^+^ and DOC content, and by indirectly altering AOA and AOB abundance.

## Discussion

### Factors influencing SA efficacy of nitrification inhibition

The efficacy of SA is mainly dependent on soil type. The differences among the three soils examined in our study may be explained by the contrasting physico-chemical properties of the soils. One of the most important among these is soil pH. The strongest and most sustained inhibitory effect of SA was found in the acidic red soil, which is consistent with previous results on other BNIs under low pH conditions, such as 1,9-decanediol, MHPP, and LN (Lu et al., 2019; Lan et al., 2022). However, SA lost its inhibitory activity in the alkaline calcareous soil. Interestingly, this pattern is opposite to that of the SNI DCD, which was more effective at suppressing nitrification in alkaline calcareous soil, with no effect in acidic red soil (**Table 2**). Several other studies have found that the efficacy of the SNIs DCD and DMPP is generally higher under more alkaline conditions (Shi et al., 2016; Bachtsevani et al., 2021; Cui et al., 2021).

These different reactivities of BNIs and SNIs under different pH regimes may be related to their interactions with ammonia oxidizer targets. Soil pH is a critical factor driving the niche partitioning of AOB and AOA (Zhang et al., 2012). Since AOA are the dominant nitrifiers in acidic red soil (Prosser et al., 2020), the strong ability of the BNIs SA and 1,9-decanediol to inhibit AOA and AOB further explains their effectiveness in acidic soil. In contrast, alkaline soils are generally considered as favorable habitats for the growth of AOB (Jia and Conrad, 2009). It is reasonable for the SNI DCD to exhibit superior inhibition in AOB-dominated alkaline soils, owing to the greater sensitivity of AOB than AOA (Shen et al., 2013). A possible reason for the loss of SA inhibition in alkaline calcareous soil is that the para-hydroxy group of SA, flanked by two methoxy groups and key to the nitrification-inhibiting effect, may react with hydroxides under alkaline conditions and become inactive in a phenoxide state (Friedman and Jürgens, 2000; Chethan and Malleshi, 2007). Thus, this leads to no effect of SA on AOB and AOA. Additionally, the degradation rates of SA under alkaline conditions might be also responsible for the disappearance of inhibition of SA, which is worthy of future determination of the SA’s dynamic concentrations in soils.

In addition, soil texture, specifically soil clay, has been shown to play a key role in affecting both efficacy and persistence of nitrification inhibitors (NIs). Generally, the sorption of NIs to the soil clays and immobilization by non-target microorganisms is linked to a decrease in the efficacy of NIs (Barth et al., 2001; Guardia et al., 2018). However, several other studies showed that the effect of clay on NIs efficacy was not only dependent on the clay proportion but also on the clay type, and the affinity of NIs to the clay (Jacinthe and Pichtel, 1992; McGeough et al., 2016). In this study, the inhibitory effect of SA was generally higher in paddy soil and red soil with higher clay content (**Figure 2B**). This is in good accordance with previous findings on the BNI 1,9-decanediol (Lu et al., 2019) and the SNI nitrapyrin combined with DMPP/DCD in several types of soil (Cui et al., 2021). It may be argued that nitrification and BNI (SA) degradation rates are likely both relatively lower in red (loamy clay) than in calcareous soil (sandy loam), hence leading to greater co-location of SA, NH_4_^+^, and ammonia-oxidizing microorganisms in time and space. Although the SNIs DCD and DMPP alone generally show reduced efficacy in soils with high clay and silt contents (Barth et al., 2008; Shi et al., 2016), they may have a stronger inhibitory effect in silt clay and clay soils in long-term incubations (Singh et al., 2008; Cui et al., 2021).

Although previous studies have found that BNIs display higher efficacy in mildly acidic soils (Lu et al., 2019; Subbarao et al., 2021; Lan et al., 2022), the present study shows that BNI efficacy might be negatively correlated with soil pH and positively correlated with soil clay content, which seems to be different from the general observation for commercial SNIs (McGeough et al., 2016; Shi et al., 2016; Bachtsevani et al., 2021; Cui et al., 2021). This finding may help identify the range of possible applications of BNIs in agricultural N management. However, it should be noted that this finding was just limited to three types of soils in China. The involving factors of SA’s inhibition merit validation in other soil types with long-term incubation periods. Continued investigations into the interactions of different soil-environmental factors (eg., factor combination pH value/clay content) in comprehensively determining the degree of inhibition of BNIs are also needed.

### Effect of SA on N_2_O emission and possible mechanisms

This is the first study to examine the effect of the BNI SA, derived from rice roots, on soil N_2_O emissions. Similar to effects seen with the BNI 1,9-decanediol (Lu et al., 2019), a strong reduction in N_2_O emission was found upon SA application in both red and paddy soils, and the reduction increased with increase in soil acidity. However, SA had no significant effect on N_2_O emission in the alkaline calcareous soil, coincident with its limited effect on the dominant AOB populations and in agreement with the inactivation of its para-hydroxyl group under alkaline conditions (Chethan and Malleshi, 2007). In AOA-dominated acidic soils, however, SA can significantly reduce N_2_O emissions, whereas DCD shows no effect (**Figure 4C**), underscoring the more dominant role in AOA in producing N_2_O emissions in acidic soils. Although our 24h closure method cannot rule out the possibility that the available oxygen in the headspace is insufficient, our estimate provides a direct linkage between N_2_O flux and soil physicochemical properties, and abundance of ammonia oxidizers treated with SA.

It is well established that ammonia oxidizers play a critical role in N_2_O emissions (Prosser et al., 2020). In the current study, SA reduced N_2_O emissions from red and paddy soils by both inhibiting AOA and AOB abundance. These dual inhibition on AOA and AOB are consistent with other identified BNIs (Lu et al., 2019; Sarr et al., 2020; Lan et al., 2022). Moreover, the inhibition of AOA by SA in red soil was higher than that of AOB (**Figures 4E, F**), indicating a higher affinity of SA for AOA than AOB, which is supported by SEM analysis, showing a stronger positive relationship between AOA and N_2_O than AOB (**Figure 5**). A previous study highlighted that AOA were more sensitive to the aromatic SNI nitrapyrin than the linear SNIs allylthiourea and DCD (Shen et al., 2013). The chemical structure of nitrification inhibitors may influence the inhibitory mechanism of ammonia monooxygenase, which may be due to the different enzyme’s active site (Wright et al., 2020) and suggests that SA may have a stronger affinity for the enzyme active sites of AOA than AOB, due to its aromatic chemical structure. In addition to the ammonia-oxidizing microorganisms, BNIs and SNIs may possess non-target effects on the rest of the soil microbiota. A recent study by Wang et al. (2021) showed that BNI sorgoleone not only inhibited the growth of a wide range of different bacterial taxa (*Flavobacterium*, *Variovorax*, *Acinetobacter*), but also stimulated the growth of certain taxa (*Nocardia* and *Methylobacillus*).

We found that application of the BNI SA significantly altered soil NH_4_^+^ and DOC, and subsequently AOA and AOB gene abundance (**Figure 5**). SEM analysis indicates that AOA abundance, not AOB, decreases significantly under increasing soil NH_4_^+^ content due to BNI SA application in paddy and red soils. This is in good agreement with studies where NH_4_^+^ substrate concentration was shown to be one of the main factors determining the abundance of AOA (Prosser and Nicol, 2012; Tao et al., 2021b). The AOA microbial community are more active under low-ammonium and other oligotrophic environments (Di et al., 2009; Verhamme et al., 2011), possibly due to higher affinities for ammonia monooxygenase of AOA (Hatzenpichler, 2012). On the contrary, higher NH_4_^+^ concentrations may inhibit the AOA abundance and activity (Verhamme et al., 2011). It should also be noted that AOA are enhanced by organic N fertilization and slow-release fertilizers, but not inorganic N fertilizers (Guo et al., 2017; Hink et al., 2018; Prosser et al., 2020). We verify that the NH_4_^+^ concentration is the key factor influencing the AOA growth in weakly acidic and acidic soils.

The SEM model further revealed that soil DOC is another factor regulating AOA and AOB abundance, since soil DOC had significantly negative correlation with AOA and AOB abundance, respectively (**Figure 5**). This is in line with other studies by Song et al. (2016) and Zhang et al. (2021), who found soil DOC content was a key factor altering the growth and community structure of ammonia oxidizer in acidic soils. Although ammonia oxidizers were traditionally believed to be strict autotrophs that are not affected by DOC, members of AOA and AOB that are more versatile could use carbon sources in a heterotrophic mode as well (Schmidt, 2009; Walker et al., 2010). In addition, soil DOC is a readily available substrate for heterotrophic microbes. These heterotrophs might produce antimicrobial compounds to suppress AOA and AOB communities, thereby competing for the ecological niche of ammonia oxidizers and shaping the larger microbial network by competitive exclusion (Jacoby and Kopriva, 2019; Wang et al., 2021). Although the mechanisms by which DOC affects ammonia oxidizers have remained still unclear, our findings show that the effect of the BNI SA on DOC is critical for the changes seen in the abundance of ammonia oxidizers, and subsequently mitigating N_2_O emissions, in weakly acidic and acidic soils. Since soil pH and clay content have great effects on nitrification (**Figure 2B**), it is also advisable to further analyze the influence of factor combination pH value/clay content on N_2_O flux in order to clarify the inhibitory process of SA more comprehensively.

### Potential applications of SA

Given the different responses of BNIs and SNIs to soil pH and texture, SA and the previously identified 1,9-decanediol present favorable alternatives to the commercially available SNIs DCD and DMPP, especially in acidic soils, which accounts for thirty percent of the earth’s ice-free lands (von Uexküll and Mutert, 1995). On the international fertilizer market, the commercial SNIs DCD and DMPP have found application in alkaline sandy loam with fast nitrification rates where AOB dominates (Jia and Conrad, 2009; Shi et al., 2016), but there has been a lack of environmentally friendly NIs suitable for acidic soils where AOA play a more significant role (Li et al., 2018; Subbarao et al., 2021). Due to spatiotemporal co-location of NH_4_^+^ and ammonia oxidizers, and strong dual inhibition of AOA and AOB, the BNI SA from rice roots has good potential as an application along with N fertilizer in acidic clay soils, if aims are to reduce N loss and alleviate soil acidification (He et al., 2012). In addition to rice and other cereal or vegetable cropping systems, plant-derived SA may also be applied to organic produce of high economic value, such as tea and blueberries, which prefer growth in acidic and NH_4_^+^-dominated soils (Britto and Kronzucker, 2002).

Of importance is also the realization that complete inhibition of soil nitrate production in soils may not be a desirable outcome, as full plant adaptation to fully reduced soil N is rare (Kronzucker et al., 1997; Kronzucker et al., 1998) and most crops suffer toxicity on pure NH_4_^+^ soil substrates (Britto and Kronzucker, 2002; Britto and Kronzucker, 2013; Li et al., 2019); BNIs, if judiciously applied, will allow for the establishment of mixed-N substrates that will allow for some nitrification to proceed (Kirk and Kronzucker, 2005), favoring plant growth and yield (Kronzucker et al., 1999; Kronzucker et al., 2000; Chen et al., 2021; Subbarao and Searchinger, 2021), while, however, greatly reducing N losses from agro-ecosystems (Coskun et al., 2017a; Coskun et al., 2017b) and the associated harmful environmental effects. Achieving such balance and avoiding the establishment of fully reduced soil environments, currently espoused by some workers in the field (Subbarao and Searchinger, 2021), must be the goal of the design of application protocols of BNIs such as SA.

The limitations of the informative value of the two-week incubation experiments of three soils without plants should be highlighted. In addition, concentrations of 100 to 500 mg SA kg^-1^ soil used in our incubation experiment are higher than SNIs and may be unacceptable for economic reasons. Therefore, great efforts must be made to reduce its applied amount while increasing the effectiveness of SA, which will contribute to promoting the actual usefulness of SA in agricultural practice. For example, the examination of BNIs synergisms, and combinations with suitable solvents or new materials will need to be taken into account to improve their stability (Lu et al., 2022). In addition to direct exogenous BNIs applications along with N fertilizers, the newly uncovered plant BNI traits could also be feasibly introduced into other crops and forage grasses as a genetic “green” mitigation strategy (Subbarao et al., 2017). By genetically exploiting the capability of crop varieties possessing high SA secretion ability from roots such as the rice genotypes identified in this study, reductions of soil nitrification and N_2_O emissions may become feasible without additional cost to or difficulties with application logistics for farmers. Deploying BNI-enabled crops could be a powerful nature-based solution to reducing N losses while maintaining yields (Subbarao et al., 2021).

## Conclusions

We provide evidence for inhibition of nitrification and N_2_O emissions by SA derived from rice roots in acidic red soil and weakly acidic paddy soil with relatively low pH and high clay percentage, and show that this may be attributable to a two-pronged inhibition of the growth of AOA and AOB microbes in soil. In contrast, our results show that SA possesses limited inhibition in an alkaline calcareous soil. The present findings reveal that soil NH_4_^+^ and DOC content are the key factors leading to the SA inhibition of AOA and AOB abundance, controlling subsequent N_2_O emissions, in paddy and red soils, and point at the possibilities of the design of novel fertilizer formulations that incorporate SA especially for acidic and AOA-dominated soils. Future studies will need to verify the inhibitory efficacy of SA and the control factors using more soil types in a longer time scale, as well as estimate the effects of SA on plant growth and diverse soil microbiota in the fields.

## Data availability statement

The original contributions presented in the study are included in the article/**Supplementary Material**. Further inquiries can be directed to the corresponding author.

## Author contributions

YL and WS designed the experiments and wrote the original draft manuscript. YL, YH, NL, WZ conducted the laboratory analysis. HK, GD and WS critically reviewed and edited the preliminary draft. All authors contributed to the article and approved the submitted version.
