# Supplementary material for: Syringic acid from rice roots inhibits soil nitrification and N2O emission under red and paddy soils but not a calcareous soil
Source: Front Plant Sci. 2022 Dec 20;13:1099689. doi: 10.3389/fpls.2022.1099689 (PMC9808040; doi:10.3389/fpls.2022.1099689)
Supplement: Supplementary file 1 [file DataSheet_1.docx]

**Supplementary methods**

***Potential nitrification activity***

Potential nitrification activity (PNA) was determined via the shaken slurry method described by Hart et al. (1994), which assess the maximum nitrate production rate of a soil sample. Briefly, fresh soil samples (15 g) were placed in Erlenmeyer flasks with 37.5 mM (NH_4_)_2_SO_4_ and phosphate buffer solution (50 mM KH_2_PO_4_; 50 mM K_2_HPO_4_; pH 7.2). The suspensions were shaken at 180 rpm for 24h on a shaker at 25°C to maintain aeration in the dark. Aliquots of 5 ml were subsequently taken at 2, 6, 12, 22, and 24 h. The aliquots were centrifuged, and the supernatants were filtered and stored at −20℃ until analysis. NO_3_^-^-N content was measured by Continuous Flow Analysis (Skalar, Breda, Netherlands), after which PNA was calculated from the rate of linear regression of nitrate contents over time (mg NO_3_^-^-N h^-1^).

Hart, S.C., Stark, J.M., Davidson, E.A., Firestone, M.K., 1994. Nitrogen mineralization, immobilization, and nitrification. In: Weaver RW, Angle JS, Bottomley BS (eds) Methods of Soil Analysis. Part 2. Microbiological and Biochemical Properties. Soil Sci. Soc. Am. J. Madison, WI, 985–1018.

**Supplementary Figures**

**Fig.S1** Relationships between AOB, AOA *amoA* gene copy numbers, and the amount of NO_3_^-^-N produced in fluvo-aquic soil (A and D), paddy soil (B and E), and red soil (C and F) after 14 days of incubation.
